# Pain Perception and Modulation: Fundamental Neurobiology and Recent Advances

**DOI:** 10.1111/ejn.70275

**Published:** 2025-10-17

**Authors:** Willem W. J. van Strien, Markus W. Hollmann

**Affiliations:** ^1^ Department of Anesthesiology, Amsterdam UMC University of Amsterdam Amsterdam the Netherlands; ^2^ Department of Anesthesiology The Netherlands Cancer Institute‐Antoni van Leeuwenhoek Hospital Amsterdam the Netherlands

**Keywords:** chronic pain, descending pathways, neuroplasticity, nociception, opioid system, pain modulation

## Abstract

Chronic pain is a major mental health burden with significant individual and societal impact. A major challenge in clinical practice is the considerable variability in treatment responses, reflecting the complexity of associated biopsychosocial factors and their neurobiological underpinnings. This narrative review presents an up‐to‐date overview of neural structures, circuits, and neurochemical systems involved in pain perception and modulation, integrating foundational and recent findings from human and animal studies. We outline current models of nociceptive processing and pain perception, emphasizing dynamic interactions between ascending nociceptive input, descending modulation, and distributed cortical networks. Additionally, we describe mechanisms at spinal, subcortical, and cortical levels, along with neuroplastic changes in chronic pain. Finally, we review key neuromodulators, including opioids, monoamines, cannabinoids, and GABA. Together, these insights support the development of personalized pain management strategies grounded in systems‐level neurobiology.

## Graphical abstract

Chronic pain is a major burden with variable treatment responses reflecting complex biopsychosocial and neurobiological factors. This narrative review synthesizes evidence on neural structures, circuits, and mediators involved in pain perception and modulation. We highlight spinal, subcortical, and cortical mechanisms, neuroplastic changes, and key neuromodulators. Created in BioRender. van Strien, W. (2025) https://BioRender.com/pcqvyj4.

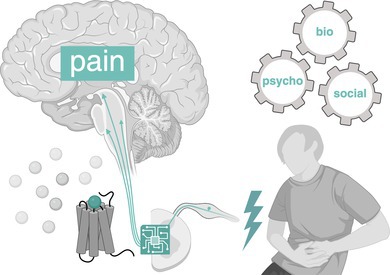

**Abbreviations**
2‐AG: 2‐arachidonoylglycerol
5‐HT: serotonin
ACC: anterior cingulate cortex
AI: anterior insula
BDNF: brain‐derived neurotrophic factor
BLA: basolateral amygdala
CB1/CB2: cannabinoid receptor type 1/type 2
CDC: Centers for Disease Control and Prevention
CeA: central nucleus of the amygdala
CGRP: calcitonin gene‐related peptide
CKK: cholecystokinin
CRF: corticotrophin‐releasing factor
dACC: dorsal anterior cingulate cortex
DBS: deep brain stimulation
DOR: δ‐opioid receptor
DRt: medullary dorsal reticular nucleus
GABA: gamma‐aminobutyric acid
IASP: International Association for the Study of Pain
ICD‐11: 11th revision of the International Classification of Diseases
KOR: κ‐opioid receptor
LC: locus coeruleus
LHb: lateral habenula
LSD: lysergic acid diethylamide
LTP: long‐term potentiation
MCC: midcingulate cortex
MOR: μ‐opioid receptor
MRGPRX: MAS‐related G protein‐coupled receptors X
NAcc: nucleus accumbens
NMDA: *N*‐methyl‐D‐aspartate
NOPR: nociceptin receptor
PAG: periaqueductal gray
PB: parabrachial complex
PCC: posterior cingulate cortex
PFC: prefrontal cortex
PI: posterior insula
PVT: thalamic paraventricular nucleus
RSC: retrosplenial cortex
RVM: rostral ventromedial medulla
S1/S2: primary/secondary somatosensory cortex
SSRIs: selective serotonin reuptake inhibitors
tDCS: transcranial direct current stimulation
TENS: transcutaneous electrical nerve stimulation
TMS: transcranial magnetic stimulation
TrkB: tyrosine kinase receptor B
VLM: ventrolateral medulla
VTA: ventral tegmental area

## 1 Introduction

Chronic pain is a leading cause of mental health burden, with towering costs (Cohen et al. 2021). Patient response to treatment varies widely, reflecting the complexity of the neurobiological systems involved and underscoring the need for a more mechanism‐based treatment approach (Cohen et al. 2021; Baron et al. 2023). Recent decades have seen considerable progress in unravelling underlying mechanisms, owing to advances in research methods such as viral‐mediated gene delivery and optogenetic control of neuronal activity in rodents, as well as improved human imaging techniques (Finnerup et al. 2021; Kuner and Kuner 2021). In this review, findings from human and animal studies are integrated into a broad overview of current understanding of the neurobiology underpinning pain perception and modulation, with a focus on the latest developments. First, fundamental concepts will be reviewed. Second, key neural structures and circuits will be discussed. Last, main neurochemical mediators will be highlighted.

## 2 Fundamental Pain Concepts: An Updated Overview

### 2.1 Nociception

Nociceptors are specialized sensory neurons that respond to actual or potential tissue damage by converting intense mechanical, thermal, and chemical stimuli into electrical signals transmitted to the dorsal horn of the spinal cord (Basbaum et al. 2009). Within the dorsal horn, this nociceptive information is processed by a complex network of interneurons before being transmitted to the brain, with the output of the dorsal horn reflecting a balance between inhibitory and excitatory activity (Finnerup et al. 2021). Disruptions to this balance, such as those caused by inflammation or nerve injury, can result in increased pain sensitivity (hyperalgesia) and pain from normally non‐painful stimuli (allodynia). Main underlying mechanisms include reduced inhibitory drive, enhanced synaptic strength termed “long‐term potentiation” (LTP), and altered signaling causing a loss of separation between non‐nociceptive and nociceptive signals (Sandkühler 2009; Kuner and Flor 2017; Finnerup et al. 2021).

**FIGURE 1:**
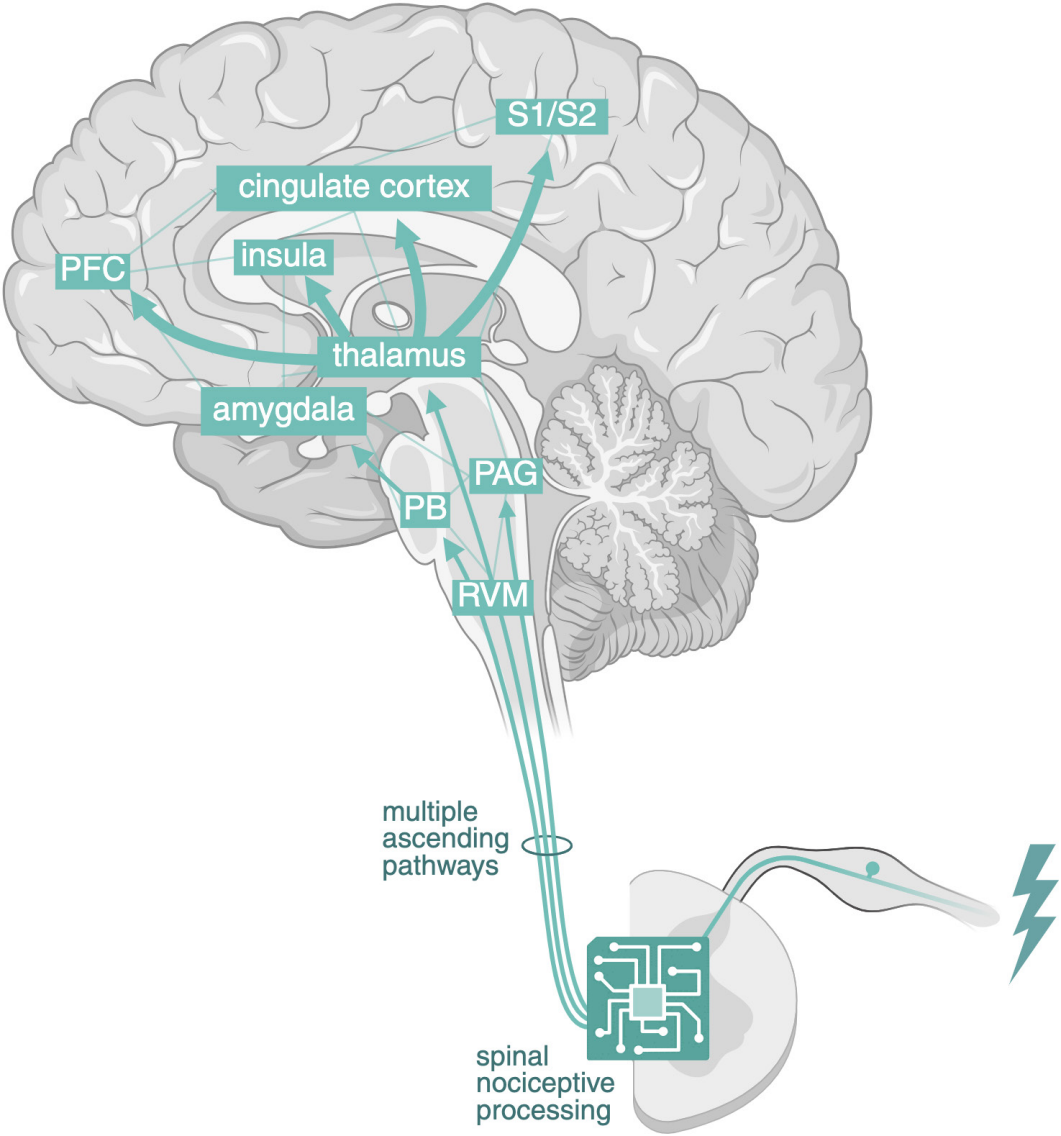
Nociception. Nociceptive information is transmitted to the dorsal horn of the spinal cord. After spinal nociceptive processing involving a complex network of interneurons, it is transmitted to the brain via multiple ascending pathways, resulting in a parallel architecture distributing nociceptive information through complex, diverse, and highly interconnected routes. A key relay is the thalamus, a highly connected hub that not only transmits but also filters and integrates sensory input through extensive cortical and subcortical interactions. Throughout these circuits, nociceptive processing is modulated by descending pathways (not shown). RVM, rostral ventromedial medulla; PB, parabrachial complex; PAG, periaqueductal gray; S1/S2, somatosensory cortices; PFC, prefrontal cortex. Created in BioRender. van Strien, W. (2025), https://BioRender.com/21yf1kj.

Nociceptive information is transmitted to the brain via multiple ascending pathways such as the spinothalamic, spinoparabrachial, and spinoreticular tracts, involving complex neural networks integrated at various levels of the nervous system (see Figure 1) (Kuner and Kuner 2021; Wang et al. 2022). This results in a parallel architecture distributing nociceptive information across multiple subcortical regions in the medulla, pons, and midbrain (Coghill 2020; Wang et al. 2022). A key relay is the thalamus, a highly connected hub that not only transmits but also filters and integrates sensory input through extensive cortical and subcortical interactions (Shine et al. 2023). Classical models distinguish three thalamocortical systems: a lateral system for sensory‐discriminative aspects of pain (mainly to the somatosensory cortices); a medial system for affective‐motivational aspects (mainly to the cingulate cortex); and a posterior system for pain intensity (mainly to the posterior insula). Newer insights, however, indicate that thalamocortical pathways are more complex and interconnected than this traditional tripartite model suggests, with pain perception often preserved even in the presence of substantial lesions (Coghill 2020; Kuner and Kuner 2021).

### 2.2 Descending Modulation

Nociceptive processing is modulated by descending pathways, which can both inhibit and facilitate nociceptive signals, affecting pain perception and behavior (Millan 2002; Finnerup et al. 2021; Bannister and Hughes 2023; De Preter and Heinricher 2024). These pathways are central to many analgesic mechanisms such as opioid‐induced analgesia (Corder et al. 2018; Bagley and Ingram 2020). They are also influenced by external factors and survival priorities, playing a key role in phenomena like the placebo effect and stress‐induced analgesia—a pain‐reduction mechanism activated in stressful situations (Millan 2002; Bravo et al. 2020; Bannister and Hughes 2023). There are several distinct yet intertwined descending control mechanisms, and it is likely that multiple of these systems are dysfunctional in patients with nociplastic pain (see Box 1) (Bannister and Hughes 2023; Kaplan et al. 2024). Main components of these pathways include the periaqueductal gray (PAG), rostral ventromedial medulla (RVM), medullary dorsal reticular nucleus (DRt), and locus coeruleus (LC) (Millan 2002; Mills et al. 2018; Costa et al. 2023; De Preter and Heinricher 2024). These structures connect with various higher brain regions contributing to descending modulation (Ossipov et al. 2010; Mills et al. 2018). Descending pathways also interact with non‐neuronal cells such as glial cells, with glial‐neuron interactions now recognized as significant contributors to (chronic neuropathic) pain (Sandkühler 2009; Kuner and Flor 2017; Malcangio and Sideris‐Lampretsas 2025).

Box 1 **Mechanistic categories of pain (Cohen et al. 2021)**

Three mechanistic categories of pain are currently distinguished:

‐ nociceptive pain, arising from actual or potential tissue damage, as signaled by nociceptors.

e.g., *trauma, osteoarthritis, inflammatory bowel disease*.

‐ neuropathic pain, arising from a lesion or disease of the somatosensory nervous system.

e.g., *peripheral neuropathy, radiculopathy, post‐stroke pain*.

‐ nociplastic pain, arising from altered nociceptive processing.

e.g., *fibromyalgia, irritable bowel syndrome, low back pain*.

However, different pain mechanisms often occur simultaneously in the same individual, termed “mixed pain.”

### 2.3 Pain Perception

Although sometimes conflated, it is important to differentiate between nociception and pain, which can occur independently. Nociception refers to the previously described neurophysiological process signaling danger, which may or may not lead to pain, while pain is not necessarily a result of nociception, for instance in the case of phantom limb pain (Baliki and Apkarian 2015; Cohen et al. 2021). Nociception often operates subconsciously, triggering automatic responses like posture adjustment to protect against injury (Baliki and Apkarian 2015; Gilam et al. 2020). Pain, on the other hand, is by definition a conscious multidimensional experience (see Box 2) (Raja et al. 2020). In other words, nociception is an input to the brain while pain is an output of the brain.

Box 2 **Current definition of pain (Raja et al. 2020).**

The International Association for the Study of Pain (IASP) revised the definition of pain in 2020 and published it along with the notes and etymology below.

Pain is “an unpleasant sensory and emotional experience associated with, or resembling that associated with, actual or potential tissue damage.”

Notes

‐ Pain is always a personal experience that is influenced to varying degrees by biological, psychological, and social factors.
‐ Pain and nociception are different phenomena. Pain cannot be inferred solely from activity in sensory neurons.
‐ Through their life experiences, individuals learn the concept of pain.
‐ A person's report of an experience as pain should be respected.
‐ Although pain usually serves an adaptive role, it may have adverse effects on function and social and psychological well‐being.
‐ Verbal description is only one of several behaviors to express pain; inability to communicate does not negate the possibility that a human or a nonhuman animal experiences pain.

Etymology:

Middle English, from Anglo‐French peine (pain, suffering), from Latin poena (penalty, punishment), in turn from Greek poine (payment, penalty, recompense).

Central to our understanding of pain perception is the biopsychosocial model. This model emphasizes that pain perception is highly subjective and shaped by many psychological and sociocultural factors (e.g., beliefs about bodily health, expectations influenced by placebo and nocebo effects, pain‐related worrying), leading to significant variability in pain perception between individuals and across situations (Baliki and Apkarian 2015; Gilam et al. 2020; Kuner and Kuner 2021).

### 2.4 Pain in the Brain

While earlier views suggested a consistent network of pain‐related brain activity dubbed the “pain matrix,” newer insights indicate that there is no specific pain network. Pain perception emerges from distributed and dynamic brain activity, with no brain region exclusively dedicated to pain processing and unique patterns underlying each individual experience (Baliki and Apkarian 2015; Kuner and Kuner 2021; Kohoutová et al. 2022). Generally speaking, there are regions primarily dealing with sensory‐discriminative aspects (such as the somatosensory cortices), while others primarily handle emotional aspects (such as the anterior cingulate cortex and anterior insula), but this distinction oversimplifies the highly interconnected networks involved in pain processing (Basbaum et al. 2009; Wiech 2016).

### 2.5 Pain and Reward

Pain serves an important evolutionary function in driving survival behavior, with both pain and pain relief linked to activity in brain reward circuits (Navratilova and Porreca 2014; Navratilova et al. 2015). These reward circuits, particularly the ventral tegmental area–nucleus accumbens pathway, enable a complex interaction between pain and pleasure, processing both aversive and rewarding signals (Leknes and Tracey 2008; Russo and Nestler 2013; Borsook et al. 2016). Although important for motivating adaptive behavior, these circuits also play a key role in maladaptive affective‐motivational changes in chronic pain and substance use disorders (Elman and Borsook 2016).

### 2.6 Chronic Pain as a Disease

**FIGURE 2:**
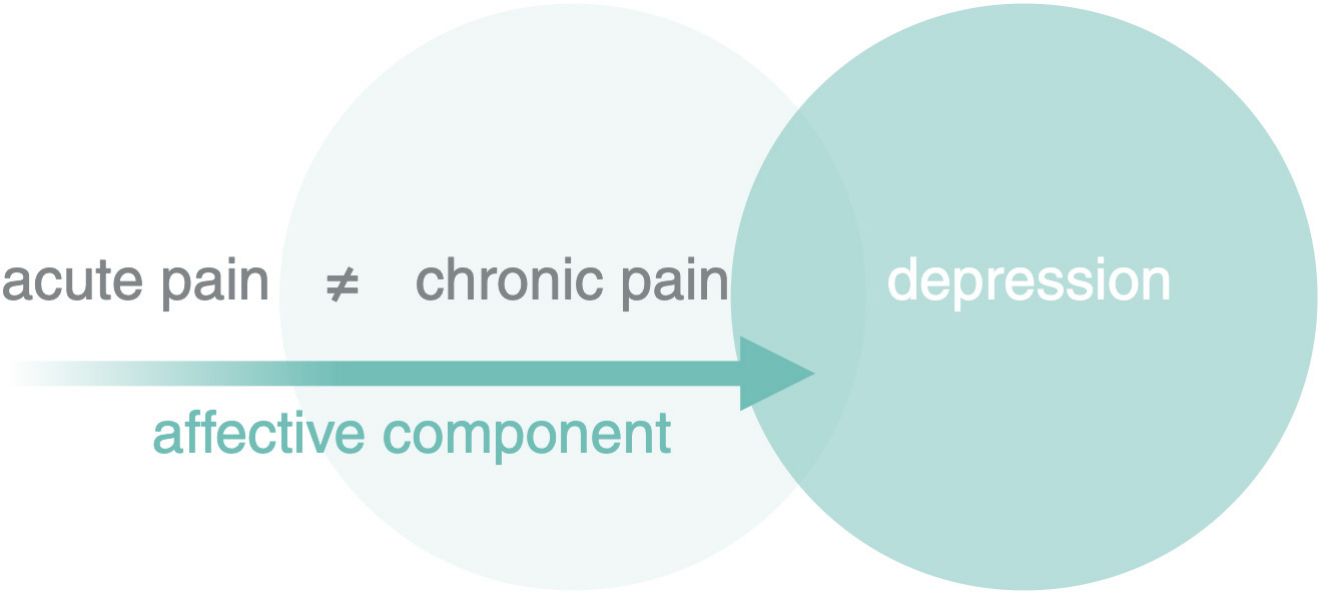
Chronic pain. Chronic pain fundamentally differs from acute pain, involving distinct neural mechanisms and brain regions, with particular emphasis on those related to affective processing. Chronic pain is a disease with notable similarities to depression. Created in BioRender. van Strien, W. (2025), https://BioRender.com/u01c0fg.

Chronic pain is defined as pain that persists or recurs for more than 3 months (Treede et al. 2019). It is a complex condition with diverse causes and presentations, ranging from fluctuating localized pain to constant widespread pain (Baron et al. 2023). It fundamentally differs from acute pain, involving distinct neural mechanisms and brain regions, with particular emphasis on those related to affective processing (see Figure 2) (Baliki and Apkarian 2015; Kuner and Flor 2017; Kuner and Kuner 2021). Accordingly, since the 11th revision of the International Classification of Diseases (ICD‐11), effective from 2022, chronic pain has been recognized as a disease in its own right (Treede et al. 2019).

Neuroplastic brain changes in chronic pain overlap considerably with those involved in depressive disorders, suggesting shared mechanisms (Bravo et al. 2020; Serafini et al. 2020; Kuner and Kuner 2021). Also, negative mood, reduced enjoyment from daily activities, and cognitive impairment—common to both depression and chronic pain—can lead to a vicious cycle of escalating depression and worsening pain (Leknes and Tracey 2008; Baliki and Apkarian 2015; Bravo et al. 2020). Chronic pain also shares many features with addiction, especially regarding reward circuitry (Elman and Borsook 2016). Furthermore, there is a strong reciprocal relationship between chronic pain and stress, with early‐life or persistent stress contributing to chronic pain, and chronic pain in turn affecting stress systems (Bravo et al. 2020).

### 2.7 Chronic Pain Mechanisms

The transition from acute to chronic pain involves extensive neuroplastic changes at molecular, synaptic, and cellular levels, resulting in altered function across systems, from nociceptors to the brain (Basbaum et al. 2009; Kuner 2010). Together, these changes contribute to pain hypersensitivity and are referred to as peripheral and central sensitization, with myriad mechanisms having been described to date, including molecular changes affecting the function and localization of important molecules such as ion channels and receptors, influencing action potential thresholds and discharge patterns; synaptic neuroplasticity driven by *N*‐methyl‐D‐aspartate (NMDA) receptor activation, causing synaptic potentiation; other changes in synaptic connections driven by, for instance, altered neurotransmitter release; and cellular changes including dendritic spine remodeling, axon degeneration, and interactions with non‐neuronal cells involved in neuronal health and neuroinflammation such as immune and glial cells (Basbaum et al. 2009; Sandkühler 2009; Kuner 2010; Kuner and Flor 2017; Finnerup et al. 2021; Malcangio and Sideris‐Lampretsas 2025).

Another important chronic pain mechanism involves imbalances in descending modulation, along with altered interactions between modulatory brainstem regions and higher brain areas such as the anterior cingulate cortex (Millan 2002; Mills et al. 2018). In chronic pain, dominant facilitatory pathways can shift the balance in spinal nociceptive processing toward increased nociceptive signaling (Kuner 2010; Finnerup et al. 2021). Additional functional and structural changes that are not measurable with current techniques are likely.

### 2.8 In Short

Nociceptors convert noxious stimuli into electrical signals that are processed in the spinal cord dorsal horn by a complex network of interneurons. Nociceptive information is transmitted to the brain via multiple ascending pathways while being modulated by several descending pathways, and reaches the cerebral cortex through various parallel routes. There, pain perception emerges from distributed and variable brain activity involving widespread regions, not necessarily related to nociceptive input. Central to pain perception is the biopsychosocial model, recognizing pain as an experience modulated by the interplay of biological, psychological, and social factors. Unlike acute pain, chronic pain is a disease in its own right, characterized by extensive neuroplastic changes and considerable overlap with depression.

## 3 Neural Structures and Circuits

### 3.1 PAG‐RVM Pathway

**FIGURE 3:**
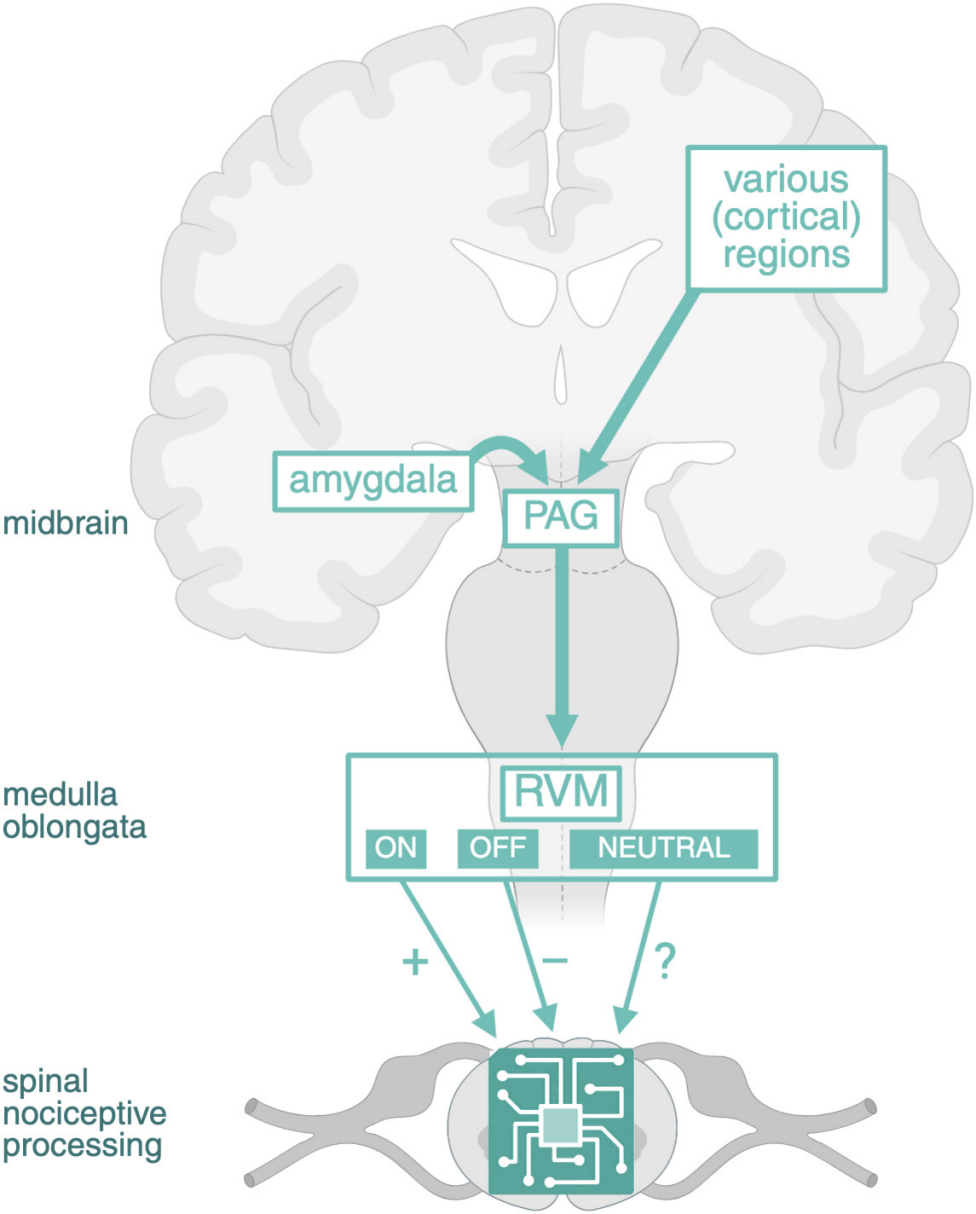
PAG‐RVM pathway. The PAG‐RVM pathway is a central descending pathway for pain modulation, receiving inputs from various cortical and subcortical regions, including the amygdala. The PAG in the midbrain projects to the RVM in the medulla oblongata, which in turn modulates spinal nociceptive processing. RVM neurons are traditionally classified into three types: ON cells facilitating nociceptive signaling, OFF cells inhibiting nociceptive signaling, and NEUTRAL cells whose role in pain modulation remains to be elucidated. PAG, periaqueductal gray; RVM, rostral ventromedial medulla. Created in BioRender. van Strien, W. (2025), https://BioRender.com/unwdpqx.

The PAG‐RVM pathway is a key descending pathway for pain modulation, involving the periaqueductal gray (PAG) in the midbrain and the rostral ventromedial medulla (RVM) in the medulla oblongata (see Figure 3) (Millan 2002; Kuner and Kuner 2021; De Preter and Heinricher 2024). The PAG is central to circuitry coordinating defensive and pain‐related behaviors, receiving inputs from diverse regions such as the amygdala and various cortical regions. It mainly sends projections to downstream targets, most importantly the RVM, which in turn modulates nociceptive processing in the dorsal horn.

RVM neurons are traditionally classified into three types: ON cells facilitating nociceptive signaling, OFF cells inhibiting nociceptive signaling, and NEUTRAL cells whose role in pain modulation remains to be elucidated (Sandkühler 2009; De Preter and Heinricher 2024). RVM projections to the spinal cord mostly use the inhibitory neurotransmitters gamma‐aminobutyric acid (GABA) or glycine, with a smaller portion being serotonergic (Ossipov et al. 2010). Although GABAergic neurons in the RVM have previously been found to facilitate nociceptive signaling by inhibiting dorsal horn interneurons that normally block nociceptive input, recent insights suggest a more nuanced role for these neurons, with both pronociceptive and antinociceptive functions (Nguyen et al. 2023). The role of serotonergic RVM neurons, located in the nucleus raphe magnus, is also under consideration. Although previously classified as NEUTRAL cells, they can have varying effects on pain, depending on activated receptor subtypes and pain context (Bardoni 2019; Kuner and Kuner 2021; Nguyen et al. 2023; De Preter and Heinricher 2024). In chronic pain, changes in RVM function—including altered ON‐ and OFF‐cell activity shaped by sensory and top‐down inputs—contribute to the persistence of pain (De Preter and Heinricher 2024).

### 3.2 Caudal Medulla

The dorsal reticular nucleus (DRt) and caudal ventrolateral medulla (VLM), adjacent to the RVM in the caudal medulla oblongata, also play significant roles in nociception and descending pain modulation (Heinricher et al. 2009; Martins and Tavares 2017). The DRt is reciprocally connected with the dorsal horn and communicates with the PAG, RVM, thalamus, amygdala, and various cortical regions (Heinricher et al. 2009; Kuner 2010; Ossipov et al. 2010). The VLM has recently been shown to modulate spinal nociceptive processing through a pathway involving the locus coeruleus (Gu et al. 2023). Together, this medullary RVM‐DRt‐VLM triad is a crucial gateway between brain and spinal cord, integrating nociceptive processing with arousal, motor, autonomic, and emotional functions (Martins and Tavares 2017).

### 3.3 Locus Coeruleus

The locus coeruleus (LC) is a small brainstem nucleus, serving as the center of the brain's noradrenergic neurotransmitter system. Its far‐reaching connections impact a wide range of functions including arousal, cognition, and motivation (Poe et al. 2020). It also plays a significant role in pain modulation, integrating information from regions including the dorsal horn, PAG, and brain areas involved in emotion and stress such as the amygdala and insula (Kuner and Kuner 2021; Suárez‐Pereira et al. 2022). A modular organization of the LC has been demonstrated, with distinct projections exerting opposing effects (Hirschberg et al. 2017). In acute pain, the LC can reduce pain through noradrenergic descending modulation, inhibiting dorsal horn projection neurons directly or indirectly through the activation of spinal inhibitory interneurons (Bravo et al. 2020; Suárez‐Pereira et al. 2022). In chronic pain, however, the LC may contribute to pain through various mechanisms including direct spinal projections, indirect descending pathways involving the DRt, and cortical projections (Bravo et al. 2020; Suárez‐Pereira et al. 2022). In particular, the LC–DRt pathway has been shown to be a key driver of persistent pain (Martins et al. 2010, 2015). The LC is also implicated in chronic pain‐related anxiety, depression, and cognitive impairment (Bravo et al. 2020).

### 3.4 Parabrachial Complex

The parabrachial complex (PB) in the pons links a wide range of sensory information to appropriate physiological and behavioral responses (Chiang et al. 2019; Palmiter 2024). It serves as a primary gateway for danger signals to higher brain areas, with neurons that express calcitonin gene‐related peptide (CGRP) acting as a general alarm system for various threats, including nociceptive information arriving via the spinoparabrachial tract (Campos et al. 2018; Palmiter 2018, 2024; Chiang et al. 2019).

In pain processing, the PB plays a central role in affective‐motivational pain dimensions, directly connecting with the central nucleus of the amygdala (CeA) and the midbrain reward system, and indirectly with the insula through a thalamic relay (Chiang et al. 2019; Raver et al. 2020; Benarroch 2022). CeA‐projecting parabrachial neurons have been shown to play a crucial role in aversive signaling and learning from painful stimuli by the subsequent formation of a threat memory (Han et al. 2015). Also, a recent study demonstrated that these neurons contribute to increased pain sensitivity after injury, with their inactivation alleviating injury‐induced pain without affecting baseline nociception (Torres‐Rodriguez et al. 2023). The PB also mediates escape responses to noxious stimuli through its connections to the reticular formation, independent of higher brain involvement (Barik et al. 2018). Additionally, it links ascending nociceptive information with descending modulatory pathways through projections to both the PAG and RVM (Chiang et al. 2019).

### 3.5 Amygdala

The amygdala is a key limbic structure central to emotional processing, particularly influencing stress, anxiety, and the affective‐motivational dimensions of pain (Bravo et al. 2020; Kuner and Kuner 2021; Neugebauer et al. 2023). It consists of two main structures critical for pain processing: the central nucleus (CeA, or “nociceptive amygdala”) and the basolateral amygdala (BLA) (Kuner 2010; Neugebauer et al. 2020). The CeA can modulate pain bidirectionally, with CeA‐mediated pain modulation primarily acting through its direct projections to the PAG, as well as through its interaction with the LC (Wilson et al. 2019; Kuner and Kuner 2021). CeA‐PAG projections play a significant role in stress‐induced analgesia, enabling appropriate behavioral responses without pain interference in painful life‐threatening situations (Kuner and Kuner 2021). Next to the CeA, the BLA also critically shapes pain experiences, with specific BLA neurons encoding negative emotional aspects of pain (Corder et al. 2019). Silencing these neurons in mice is sufficient to reduce pain‐related affective‐motivational behaviors without affecting the detection of noxious stimuli, underscoring the BLA's role in transforming nociception into an emotional response necessary for adaptive pain behaviors (Corder et al. 2019).

### 3.6 Thalamus

The thalamus, located in the diencephalon, is a small but extensively connected structure affecting a broad range of cognitive and behavioral functions (Shine et al. 2023). Recent neuroimaging advances have transformed our understanding of the thalamus from being just a relay station to an active coordinator of brain dynamics, acting as a crucial hub that integrates multimodal information across cortical networks (Shine et al. 2023). In pain processing, the thalamus is essential for transmitting, filtering, and processing sensory information (Kuner 2010; Kuner and Kuner 2021). It also receives substantial cortical feedback, modulating both acute and chronic pain (Kuner and Kuner 2021). A significant component of this modulatory function is the thalamic paraventricular nucleus (PVT), which is part of a neural circuit with the CeA and PAG. This PVT‐CeA‐PAG circuit has been implicated in neuropathic pain and persistent pain conditions (Liang et al. 2020). Also, in a mouse model of neuropathic pain, it was recently found that a PVT‐BLA pathway is important in chronic pain‐induced anxiety, with its manipulation affecting both pain and anxiety (Tang et al. 2023).

### 3.7 VTA‐NAcc Reward Circuit

The ventral tegmental area (VTA) and nucleus accumbens (NAcc) are central to the brain's reward system, which influences affective‐motivational aspects of pain and has been linked to chronic pain‐related depression and addiction (Russo and Nestler 2013; Bravo et al. 2020; Serafini et al. 2020). The VTA and NAcc are intricately connected and receive inputs from various regions including the amygdala, PB, lateral habenula, anterior cingulate cortex, and prefrontal cortex, influencing reward and aversion responses (Russo and Nestler 2013; Navratilova and Porreca 2014; Borsook et al. 2016; Benarroch 2022). In chronic pain, changes in functional connectivity between the prefrontal cortex and NAcc affect how pain and pain relief are perceived and processed (Baliki and Apkarian 2015; Kuner and Kuner 2021).

### 3.8 Lateral Habenula

The lateral habenula (LHb), in the dorsal thalamus, is often referred to as the brain's “antireward center” as it processes negative reward signals such as punishment, disappointment, and the omission of expected rewards. It integrates a broad range of inputs, primarily from limbic and basal ganglia systems, influencing motivational, cognitive, and motor processes (Hu et al. 2020). The LHb appears to be involved in pain modulation through its connections to the PAG and raphe nuclei and is essential for comorbid depressive symptoms in chronic pain (Tappe‐Theodor and Kuner 2019; Kuner and Kuner 2021). In chronic pain, the LHb becomes more active, affecting serotonergic neurons that project to several cortical and limbic structures involved in cognitive and emotional functions, as well as to the spinal cord via descending pathways, resulting in context‐dependent modulation of spinal nociceptive processing (Tappe‐Theodor and Kuner 2019; Zhou et al. 2019).

### 3.9 Hippocampus

The hippocampus, known for its role in learning and memory, has also been implicated in pain perception and modulation, linking memory and the emotional value of stimuli potentially through its connections to the VTA and NAcc (Russo and Nestler 2013; Kuner and Kuner 2021). In chronic pain patients, the hippocampus displays changes similar to depression, including reductions in volume and neuroplasticity as well as altered connectivity with the prefrontal cortex, leading to cognitive and emotional impairments (Bravo et al. 2020; Kuner and Kuner 2021).

### 3.10 Somatosensory Cortex

Both the primary (S1) and secondary (S2) somatosensory cortices have long been ascribed fundamental roles in processing pain‐related information, with S1 primarily involved in pain localization and S2 focusing on the intensity of painful stimuli (Bushnell et al. 1999). However, newer insights suggest that this is an oversimplification and that their role is more multifaceted, with factors like emotions impacting S1 processing and S2 modulating depression and pain comorbidity (Tan and Kuner 2021). Also, manipulating S1 activity can alter pain sensitivity, aversive avoidance behaviors, and activity in other pain‐related regions such as the amygdala, anterior cingulate cortex, and insula (Tan et al. 2019).

### 3.11 Cingulate Cortex

The cingulate cortex, encircling the corpus callosum, can be divided into four regions: anterior cingulate cortex (ACC), midcingulate cortex (MCC), posterior cingulate cortex (PCC), and retrosplenial cortex (RSC). This division, based on cytoarchitecture and connectivity, differs from earlier models that identified the MCC as the dorsal ACC (dACC) (Vogt 2005; Shackman et al. 2011). Particularly, the ACC and MCC have important roles in pain processing.

The ACC is a central hub for sensory, emotional, and cognitive processes, playing a crucial role in both the sensory and especially emotional dimensions of pain (Shackman et al. 2011; Tan and Kuner 2021; Journée et al. 2023). Nociceptive information reaches the ACC through pathways from the thalamus (via the spinothalamic tract), amygdala (via the spinoparabrachial tract), and pain‐related cortical regions such as the somatosensory cortex and insula (Bliss et al. 2016). ACC neurons project to various subcortical and cortical regions, including the PAG, LC, hypothalamus, amygdala, and prefrontal cortex, contributing to its complex role in pain processing and emotional responses (Bliss et al. 2016). ACC neurons also project directly to the dorsal horn, influencing nociceptive processing independently of brainstem pathways and likely contributing to central sensitization in chronic pain (Bliss et al. 2016; Chen et al. 2018). Furthermore, the ACC is implicated in anxio‐depressive consequences of chronic pain (Barthas et al. 2015; Journée et al. 2023). In chronic pain patients, increased ACC activity is linked to increased pain perception and negative emotional factors (Tan and Kuner 2021).

The MCC processes affective‐motivational components of pain and manages pain‐related behaviors, connecting to motor centers responsible for emotional behavior (Shackman et al. 2011). It integrates negative affect, pain, and cognitive control, with consistent activation of a common region within the MCC across these domains, challenging the traditional notion of a strict cognitive versus affective division in the cingulate cortex (Shackman et al. 2011). The MCC also mediates increased pain sensitivity, particularly through its connection with the posterior insula, contributing to sustained hypersensitivity through facilitatory serotonergic projections to the spinal cord (Tan et al. 2017).

### 3.12 Insula

The insular cortex, or “insula,” located within the lateral sulcus, is an integration hub connecting numerous cortical and subcortical regions. It integrates sensory inputs from various external and internal modalities, playing a role in a wide range of functions including sensory, emotional, motivational, and cognitive processes (Gogolla 2017; Benarroch 2019). In humans, the insula is divided into anterior (AI) and posterior (PI) lobes. The PI receives multimodal sensory inputs, processes them, and relays them to the AI. The AI further processes this information and interacts with areas involved in cognitive and emotional control. This allows the insula to act as an interface between bodily sensations and emotions, contributing to perceptual awareness, social behavior, and decision‐making (Benarroch 2019).

In pain processing, nociceptive input flows from the PI to the AI (Tan and Kuner 2021). The PI is particularly involved in processing sensory aspects, such as its intensity, location, and modality, and it consistently activates in response to noxious stimuli (Tan and Kuner 2021). Although the PI is important for the somatosensory component of pain, it does not have a critical role in encoding anxio‐depressive consequences of chronic pain (Barthas et al. 2015). The AI, on the other hand, interacts with the prefrontal cortex and integrates emotional and affective processes (Tan and Kuner 2021). Also, a recent study demonstrated that while the PI mirrors the actual intensity of constant nociceptive stimuli, the AI and amygdala are key in the transition from nociception to pain perception, significantly affecting the subjective experience of intensity (Gélébart et al. 2023). The AI's importance in linking sensory experiences with emotional responses is evident in conditions like ‘pain asymbolia,’ where patients with insular lesions can recognize pain but lack negative emotional reactions (Gogolla 2017). The AI is also important for empathy, activating both when experiencing pain and when observing pain in others (Gogolla 2017). Last, injury‐triggered long‐term potentiation and plasticity in the insula are important factors in the development and maintenance of chronic pain (Tan and Kuner 2021).

### 3.13 Prefrontal Cortex

The prefrontal cortex (PFC) is involved in a wide range of functions, including cognition, executive function, and emotion. It also plays a crucial role in pain perception and modulation, with changes in its activity and functional connectivity significantly impacting pain and associated comorbidities (Shiers and Price 2020; Tan and Kuner 2021). A recent study in healthy volunteers showed intraindividual pain variability during repeated noxious stimuli to be associated with dorsolateral PFC activity and connectivity, possibly reflecting a PFC‐mediated attentional effect on pain perception (Crawford et al. 2023).

In chronic pain, emerging evidence suggests reduced PFC activity in patients, potentially contributing to pain persistence, possibly due to disrupted descending analgesic circuitry (Shiers and Price 2020). Human neuroimaging studies highlight a key PFC‐PAG pathway enabling top‐down control of pain modulation, while reduced activity in this pathway in mice is linked to persistent neuropathic pain (Tan and Kuner 2021). Building on this, a recent study identified PFC neurons that exclusively project to the PAG and change in chronic pain, with reduced activity leading to increased pain due to impaired descending inhibition (Bhattacherjee et al. 2023). In chronic pain patients, there is also a loss of fiber track density and altered white matter connectivity from the PFC to other pain processing regions, such as the insula and anterior cingulate cortex, suggesting disrupted PFC output (Shiers and Price 2020). Chronic pain has also been shown to cause changes in functional PFC connectivity correlating with pain intensity and duration, altering pain perception and impacting pain‐related anxiety and depression (Bravo et al. 2020; Serafini et al. 2020). Interestingly, a recent mouse study demonstrated that long‐term fear memories are stored in specific PFC neurons to shape subsequent pain perception, and silencing these neurons can alleviate chronic pain (Stegemann et al. 2023).

### 3.14 Lesion Studies

Lesion studies reveal how damage to specific brain regions alters nociceptive processing and pain perception, providing insights into the contribution of distinct neural structures. Thalamic strokes frequently result in central post‐stroke pain, underscoring the thalamus as a key relay for both sensory and affective dimensions (Klit et al. 2009). Insular lesions can disrupt intensity coding and, in some cases, produce pain asymbolia, where nociceptive stimuli are detected but not experienced as unpleasant (Gogolla 2017; Benarroch 2019). Damage to the anterior cingulate cortex reduces the affective‐motivational component of pain while sparing sensory‐discriminative aspects, highlighting its role in emotional modulation (Shackman et al. 2011; Journée et al. 2023). More recently, lesion‐network mapping has shown that distinct pain syndromes converge on shared networks rather than isolated sites, emphasizing the importance of connectivity in pain perception (Rosner et al. 2023).

### 3.15 Neuromodulation

Neuromodulation studies demonstrate how direct modulation of neural circuits can influence pain, providing evidence for the involvement of specific neural regions. Non‐invasive methods such as transcranial magnetic stimulation (TMS) and transcranial direct current stimulation (tDCS) over motor and prefrontal cortices implicate thalamo‐cortical and frontolimbic networks in pain modulation, though clinical effects remain modest (O'Connell et al. 2018; Soliman et al. 2025). Transcutaneous electrical nerve stimulation (TENS) is widely used and thought to reduce nociceptive transmission via inhibitory circuits, with evidence mixed but suggesting potential benefit (Johnson et al. 2015; Knotkova et al. 2021; Soliman et al. 2025). Spinal cord and dorsal root ganglion stimulation have the strongest clinical evidence among neuromodulation techniques, providing benefit across several pain syndromes, while deep brain stimulation of the thalamus, ACC, and PAG remains experimental with variable results (Knotkova et al. 2021). Together, these approaches illustrate the distributed nature of pain circuits and suggest that targeted interventions have the potential to reshape them.

### 3.16 Psychedelics

Psychedelic compounds such as psilocybin and LSD have attracted attention for their potential to modulate brain circuits relevant to pain. Acting primarily through 5‐HT2A receptors, they appear to alter connectivity across thalamo‐cortical and frontolimbic networks, loosening rigid predictive processing and influencing sensory–affective integration involved in pain perception (Castellanos et al. 2020; van Elk and Yaden 2022; Erritzoe et al. 2024). Psychedelics have also been associated with neuroplastic changes that could support longer‐term adjustments in networks implicated in pain modulation, although mechanisms remain incompletely understood (Siegel et al. 2024). Clinical evidence is still preliminary, but early findings suggest potential benefits in pain conditions (Goel et al. 2023).

### 3.17 In Short

The PAG‐RVM pathway, caudal medulla, and locus coeruleus are central to descending pain modulation. The parabrachial complex is a primary gateway for danger signals including nociception and contributes to aversive learning and affective‐motivational aspects of pain. The amygdala critically shapes emotional dimensions of pain. The thalamus is not just a relay station but a central hub integrating cortical and subcortical inputs. The VTA–NAcc reward circuit links pain and reward processes. The lateral habenula encodes aversive “antireward” signals relevant to pain. The hippocampus is involved in memory‐related pain processing. The role of the somatosensory cortices extends beyond localization and intensity coding to include emotional influences. The cingulate cortex integrates sensory and emotional aspects of pain and influences pain sensitivity through extensive connections. The insula is an integration hub for sensory, emotional, and cognitive processes. The prefrontal cortex is critical for pain perception, with changes in its activity and connectivity affecting pain and associated emotional states, especially in chronic pain. Lesion studies, neuromodulation, and psychedelics highlight how specific brain circuits contribute to pain perception and modulation, extending anatomical insights toward therapeutic relevance.

## 4 Neurochemical Mediators

### 4.1 Opioid System

The opioid system includes four G protein–coupled receptors found throughout the nervous system: the μ‐opioid receptor (MOR), δ‐opioid receptor (DOR), κ‐opioid receptor (KOR), and nociceptin receptor (NOPR) (Corder et al. 2018; Che and Roth 2023; Costa et al. 2023). These receptors interact with four main endogenous agonists with differing receptor specificities: β‐endorphins (primarily on MOR, also on DOR), enkephalins (on MOR and DOR), dynorphins (primarily on KOR), and nociceptin (exclusively on NOPR) (Corder et al. 2018; Kuner and Kuner 2021; Che and Roth 2023). In recent years, other atypical opioid receptors have been discovered, such as the MRGPRXs, which are expressed in primates but not in mice or rats (Che and Roth 2023). Adding complexity, opioid receptors exist as multiple isoforms and can form heteromers with other opioid and non‐opioid receptors, affecting ligand binding and signaling (Costa et al. 2023; Gaborit and Massotte 2023; Varga et al. 2023). Recent advances suggest a significant role for these isoforms and heteromers, particularly in chronic pain and opioid therapy (Costa et al. 2023; Gaborit and Massotte 2023).

Activation of opioid receptors typically suppresses neural function, impacting pain and reward pathways (Corder et al. 2018). Most current opioid analgesics target MOR, providing pain relief but also causing adverse effects such as constipation, respiratory depression, and the potential for abuse (Traynor and Moron 2023; Varga et al. 2023). Agonists targeting KOR, DOR, and NOPR also have analgesic actions, while varying in their side effects (Varga et al. 2023). Hence, there is an ongoing effort to develop novel drugs targeting multiple receptors simultaneously to provide pain relief with fewer adverse effects (Varga et al. 2023). Other innovative approaches include biased opioid ligands favoring specific downstream signaling cascades and comedication to finetune the effects of opioid agonists—although without significant clinical breakthroughs yet (Corder et al. 2018; Costa et al. 2023; Traynor and Moron 2023; Varga et al. 2023).

Central to opioid analgesia are descending modulatory pathways involving the PAG and RVM, ultimately inhibiting spinal nociceptive processing (Corder et al. 2018; Bagley and Ingram 2020). Opioid‐induced disinhibition of PAG neurons projecting to the RVM activates descending pathways (Lau et al. 2020; Winters et al. 2022). Opposing this PAG‐RVM pathway is a PAG‐LC pathway, where opioids are now recognized to suppress descending inhibition, counteracting opioid analgesia (Kim et al. 2018). Another significant opioid pathway involves the DRt, where endogenous opioids are released by local interneurons and enkephalinergic fibers from other brain areas, with MOR activation in the DRt leading to pain relief through inhibition of descending facilitatory neurons (Costa et al. 2023). However, the exact roles and regulation of different endogenous opioids in descending modulation, especially in the context of chronic pain, are not yet fully understood (Bagley and Ingram 2020). Another important brain region rich in opioid receptors is the amygdala, although the precise role of amygdala opioid signaling in pain remains to be uncovered (Neugebauer et al. 2023). It has been found, however, that CeA activity is regulated by dynorphin, with dynorphin inducing aversion and dysphoria, in contrast to other endogenous opioids (Kuner and Kuner 2021). Also, MOR agonists have been shown to relieve pain by disinhibiting CeA neurons projecting to the PAG (Bagley and Ingram 2020). Last, opioid receptors are also abundant in cortical areas, including the cingulate cortex (Tan and Kuner 2021). Opioid signaling in the cingulate cortex plays a critical role in pain modulation, with opioid ACC circuits involved in bidirectional pain modulation (Navratilova et al. 2023; Neugebauer et al. 2023).

**FIGURE 4:**
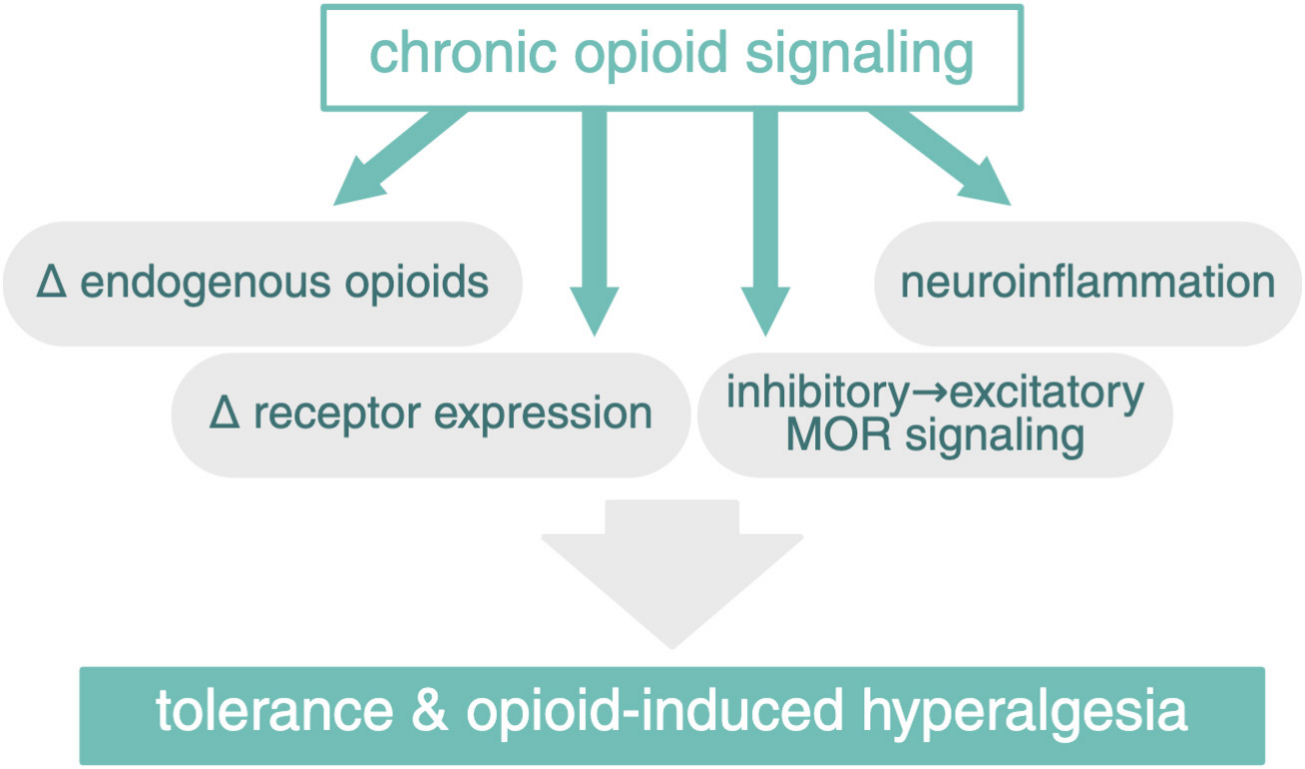
Chronic opioid signaling. Chronic opioid signaling can lead to changes in endogenous opioids, altered receptor expression, neuroinflammation, and a shift from inhibitory to excitatory MOR signaling, leading to tolerance and opioid‐induced hyperalgesia. MOR, μ‐opioid receptor. Created in BioRender. van Strien, W. (2025), https://BioRender.com/szjn7pr.

Chronic pain is associated with increased endogenous opioid levels, leading to chronic opioid signaling akin to long‐term opioid drug exposure (Ballantyne 2018; Costa et al. 2023). Chronic opioid signaling can lead to changes in the endogenous opioid system, including altered receptor expression, neuroinflammatory responses, and even a potential shift from inhibitory to excitatory MOR signaling, leading to opioid analgesic tolerance and opioid‐induced hyperalgesia (see Figure 4) (Ballantyne 2018; Corder et al. 2018; Costa et al. 2023). This explains why long‐term opioid therapy in chronic pain patients may not improve pain or pain‐related function and has been associated with more adverse effects compared to non‐opioid medications (Krebs et al. 2018). This is reflected in the 2022 Centers for Disease Control and Prevention (CDC) guidelines discouraging opioid drug initiation for common types of (chronic) pain and advising careful weighing of benefits and risks when prescribing opioids in exceptional cases (Dowell et al. 2022).

### 4.2 Noradrenergic System

The noradrenergic system modulates pain through both descending and ascending pathways, as well as peripheral effects. Descending pathways, originating from the locus coeruleus and other noradrenergic brainstem cell groups, have traditionally been known to inhibit spinal nociceptive processing, directly via actions on α_2_‐adrenoceptors and indirectly via α_1_‐adrenoceptor‐mediated activation of inhibitory interneurons (Millan 2002; Bannister et al. 2009; Ossipov et al. 2010; Pertovaara 2013). Current evidence, however, indicates that noradrenergic influence can be both inhibitory and facilitatory, particularly in chronic pain (Kuner and Kuner 2021; Suárez‐Pereira et al. 2022). This could be related to neuroimmune interactions, with α_1_‐adrenoceptor‐expressing astrocytes having been identified as important regulators of noradrenergic signaling in the dorsal horn (Kohro et al. 2020). Also, ascending noradrenergic pathways, projecting to areas such as the thalamus, amygdala, and various cortical regions, are increasingly implicated in heightened pain, especially in chronic pain (Kuner and Kuner 2021). Furthermore, noradrenergic pathways interact with a wide range of neurochemical mediators including opioids, serotonin, dopamine, and endocannabinoids, adding to their complexity (Millan 2002).

Paradoxically, despite the potential for noradrenergic signaling to increase pain, noradrenergic drugs like duloxetine and amitriptyline are standard chronic pain treatments, underscoring the complexity of the underlying neural circuits with potentially opposing effects (Kuner and Kuner 2021). In fact, noradrenergic mechanisms significantly contribute to the analgesic effects of duloxetine and amitriptyline, which work through both acute central α_2_‐adrenoceptor mechanisms and delayed peripheral β_2_‐adrenoceptor‐mediated neuroimmune processes (Kremer et al. 2018). Also, these noradrenergic mechanisms appear to be more important for pain relief than serotonergic mechanisms (Bannister et al. 2009; Kuner and Kuner 2021).

### 4.3 Serotonergic System

Descending serotonergic projections, mainly originating from the brainstem dorsal raphe nucleus and nucleus raphe magnus in the RVM, play a significant role in descending pain modulation (Millan 2002; Kuner and Kuner 2021). These brain regions send serotonergic fibers to the spinal cord, where they influence nociceptive processing through a variety of serotonin receptors, particularly the 5‐HT_3_ receptor subtype (Bannister et al. 2009; Sandkühler 2009; Kuner and Kuner 2021). To date, 14 serotonin receptor subtypes have been identified, through which serotonin is able to exert both inhibitory and facilitatory effects, depending on activated receptor subtypes and pain context (Bannister et al. 2009; Bardoni 2019; Kuner and Kuner 2021). Also, a recent study in a neuropathic pain model demonstrated that a dysregulated spinal chloride transporter transforms serotonin‐mediated modulation from inhibitory to facilitatory, confirming that serotonin circuits can have variable effects based on local spinal conditions (Aby et al. 2022). This complex action explains why pure serotonergic drugs like selective serotonin reuptake inhibitors (SSRIs) have shown limited efficacy in chronic pain treatment (Bardoni 2019). Serotonin receptors play a crucial role in mediating central sensitization, with pain facilitation through 5‐HT_3_ receptors being prevalent in neuropathic pain conditions (Bardoni 2019; Kuner and Kuner 2021). Furthermore, newer findings highlight a vast ascending network of serotonergic projections from the dorsal raphe nucleus to various areas of the brain, playing a role in emotional reactions to aversive stimuli and offering new opportunities for research in chronic pain conditions (Kuner and Kuner 2021). Compared to the complex actions in the central nervous system, the action of serotonin in peripheral tissue is relatively simple, with injury inducing its release, activating, and sensitizing nociceptors (Bannister et al. 2009).

### 4.4 Dopaminergic System

The dopaminergic system is integral to both pain and pleasure, including supraspinal reward circuits as well as descending projections that modulate spinal nociceptive processing (Millan 2002; Leknes and Tracey 2008). This system facilitates learning and motivates behavior (Navratilova et al. 2015). Dopamine receptors are categorized into two types: D_2_‐like receptors (D_2_, D_3_, D_4_), which typically reduce neuronal activity, and D_1_‐like receptors (D_1_, D_5_), which increase it (Millan 2002). Key components of the dopaminergic system include the VTA and the NAcc. Dopaminergic neurons in the VTA show varied responses to painful stimuli, with some being inhibited and others excited by acute noxious stimuli (Benarroch 2022). Their exact roles are not fully understood due to the functional heterogeneity of these neurons, their varied targets, differential responses to painful stimuli, neuroplastic changes in response to nerve injury, and effects on different brain areas involved in emotion, reward processing, and motivation (Serafini et al. 2020; Benarroch 2022). A key inhibitory influence on VTA dopaminergic neurons comes from the lateral habenula when encountering aversive stimuli (Benarroch 2022). In chronic pain, rodent and human studies suggest a hypodopaminergic state characterized by altered dopamine levels and metabolism, with reduced dopaminergic transmission in the VTA‐NAcc reward circuit (Serafini et al. 2020; Benarroch 2022). Of particular interest is pain in Parkinson's disease, which—while involving complex, multi‐level mechanisms—is closely linked to the dopaminergic degeneration characteristic of the condition (Lei et al. 2024).

### 4.5 Cannabinoid System

The cannabinoid system regulates various biological processes, including neurodevelopment, neurotransmitter release, synaptic plasticity, and cytokine release from microglia (Cristino et al. 2020). It consists of cannabinoid receptors 1 and 2 (CB_1_ and CB_2_), the two endocannabinoids anandamide and 2‐arachidonoylglycerol (2‐AG), and related metabolic enzymes (Cristino et al. 2020). CB_1_ receptors are important for synaptic plasticity and various brain functions, impacting neurotransmission, metabolism, and cell differentiation (Cristino et al. 2020). CB_2_ receptors mainly regulate immune responses, particularly through their presence in microglia affecting cytokine release, while also influencing neurogenesis, blood–brain barrier permeability, and possibly neuronal excitability (Cristino et al. 2020). Cannabinoids exhibit pain‐relieving effects at multiple levels of the nervous system, directly inhibiting spinal nociceptive processing as well as interacting with various neurotransmitters and supraspinal pain modulatory pathways (Millan 2002; Sirucek et al. 2023). Endocannabinoids are involved in stress‐induced analgesia through engagement of the PAG, and injection of cannabinoids into the PAG relieves pain (Sirucek et al. 2023). However, besides acting on CB_1_ and CB_2_, cannabinoids interact with a range of non‐cannabinoid receptors and ion channels, displaying a complex pharmacology that poses challenges for effective and safe targeting, requiring innovative drug development strategies like biased signaling and multitarget approaches to maximize therapeutic benefits while minimizing (psychoactive) side effects (Cristino et al. 2020; Morales and Jagerovic 2020). Despite this broad biological relevance, recent recommendations advise against cannabinoid treatment for neuropathic pain due to limited efficacy and tolerability (Soliman et al. 2025).

### 4.6 GABAergic System

The GABAergic system exerts inhibitory control that is essential for maintaining the balance between excitation and inhibition, coordinating neural activity within and across brain networks (Caputi et al. 2013). Altered GABAergic signaling has been linked to changes in both the sensory and emotional dimensions of pain, reflecting its role in integrating sensory, affective, and descending control mechanisms (Kuner and Kuner 2021). At the supraspinal level, GABAergic neurons exert complex, bidirectional influences on pain modulation, while in the spinal cord GABA provides critical inhibitory control of nociceptive processing (Millan 2002; Kuner and Kuner 2021).

### 4.7 Other Key Molecules

Other key molecules include other classical neurotransmitters such as glutamate, glycine, and acetylcholine, which are all involved in pain perception and modulation throughout the central nervous system (Millan 2002; Sandkühler 2009; Kuner and Kuner 2021; Sirucek et al. 2023; Sullere et al. 2023). In addition, there are many relevant neuropeptides involved, with their actions complimenting the classical neurotransmitters. Prominent neuropeptides in pain perception and modulation include: calcitonin gene‐related protein (CGRP), affecting nociceptor sensitization, synaptic plasticity, and affective‐motivational aspects; substance P, with a central role in spinal nociceptive transmission; oxytocin, modulating pain through interactions with opioid and cannabinoid systems; orexin peptides, that interact with endocannabinoid signaling and reward circuits; galanin, interacting with descending opioid and monoaminergic pathways; neurotensin, with various supraspinal effects and primarily inhibitory spinal effects; cholecystokinin (CKK), with pronounced anti‐opioid effects; and corticotrophin‐releasing factor (CRF), a key coordinator of stress responses, increasing arousal and vigilance through its effects on the locus coeruleus (Millan 2002; Russo and Nestler 2013; Ballantyne 2018; Neugebauer et al. 2020; Kuner and Kuner 2021). Also noteworthy is brain‐derived neurotrophic factor (BDNF)‐tyrosine kinase receptor B (TrkB) signaling, with varying influences on pain processing and an important role in opioid‐induced hyperalgesia (Basbaum et al. 2009; Kuner 2010; Lim and Cengiz 2022).

### 4.8 In Short

The opioid system includes four receptors (μ, δ, κ, and nociceptin) and endogenous agonists, mainly providing pain relief through descending modulatory pathways. Opioid drugs acting on this system are potent analgesics but have significant adverse effects, with chronic pain and opioid drugs posing risks of opioid tolerance and opioid‐induced hyperalgesia. Noradrenergic and serotonergic systems modulate pain through diverse neural circuits and receptor subtypes, sometimes with opposing effects. The dopaminergic system, involved in pain and pleasure, modulates pain via descending pathways and supraspinal reward circuits, with chronic pain affecting dopamine transmission. The cannabinoid system, essential for neurodevelopment and neuroimmune responses, is involved in pain perception and modulation but faces drug development challenges due to its complex pharmacology. The GABAergic system provides key inhibition at spinal and supraspinal levels, balancing excitation and inhibition in pain modulation. Other key molecules include classical neurotransmitters like glutamate, glycine, and acetylcholine, as well as neuropeptides such as CGRP, substance P, oxytocin, orexin, galanin, neurotensin, CKK, and CRF, which interact with various pathways related to pain perception and modulation.

## 5 Concluding Remarks

Pain neuroscience is rapidly advancing, with this review providing an updated overview of the neurobiology underpinning pain perception and modulation. Several limitations are important to note. First, much of our understanding of pain mechanisms comes from rodent research. Although there are many commonalities between rodents and humans, genetic and functional differences render animal models unable to fully capture the complexity of human pain (Sadler et al. 2022; Baron et al. 2023). Second, although chronic pain is most prevalent in women, preclinical studies primarily involve male rodents, establishing a bias in current knowledge (Mogil 2020). Last, a fundamental limitation is that pain is by definition a multidimensional personal experience, and neurobiological findings may not always translate to real‐world individual pain perception and clinical practice.

Currently, a major challenge in clinical practice is the high variability in pain treatment responses. Future work should prioritize the development of personalized pain treatments that build on the systems‐level neurobiology outlined in this review.

## Author Contributions

WvS and MH developed the conceptual framework. WvS prepared the original draft and figures, while MH supervised the work. Both authors contributed to reviewing and editing the manuscript and approved the final version.

## Conflicts of Interest

The authors declare no competing interests.

## Peer Review

The peer review history for this article is available at https://www.webofscience.com/api/gateway/wos/peer‐review/10.1111/ejn.70275.

## References

[ejn70275-bib-0001] Aby, F. , L.‐E. Lorenzo , Z. Grivet , et al. 2022. “Switch of Serotonergic Descending Inhibition into Facilitation by a Spinal Chloride Imbalance in Neuropathic Pain.” Science Advances 8: 1–18. 10.1126/sciadv.abo0689 PMC9328683 35895817

[ejn70275-bib-0002] Bagley, E. E. , and S. L. Ingram . 2020. “Endogenous Opioid Peptides in the Descending Pain Modulatory Circuit.” Neuropharmacology 173: 108131. 32422213 10.1016/j.neuropharm.2020.108131 PMC7313723

[ejn70275-bib-0003] Baliki, M. N. , and A. V. Apkarian . 2015. “Nociception, Pain, Negative Moods, and Behavior Selection.” Neuron 87: 474–491. 26247858 10.1016/j.neuron.2015.06.005 PMC4529956

[ejn70275-bib-0004] Ballantyne, J. C. 2018. “The Brain on Opioids.” Pain 159: S24–S30. 30113944 10.1097/j.pain.0000000000001270

[ejn70275-bib-0005] Bannister, K. , L. A. Bee , and A. H. Dickenson . 2009. “Preclinical and Early Clinical Investigations Related to Monoaminergic Pain Modulation.” Neurotherapeutics 6: 703–712. 19789074 10.1016/j.nurt.2009.07.009 PMC5084291

[ejn70275-bib-0006] Bannister, K. , and S. Hughes . 2023. “One Size Does Not Fit All: Towards Optimising the Therapeutic Potential of Endogenous Pain Modulatory Systems.” Pain 164: e5–e9. 35594517 10.1097/j.pain.0000000000002697 PMC9756434

[ejn70275-bib-0007] Bardoni, R. 2019. “Serotonergic Modulation of Nociceptive Circuits in Spinal Cord Dorsal Horn.” Current Neuropharmacology 17: 1133–1145. 31573888 10.2174/1570159X17666191001123900 PMC7057206

[ejn70275-bib-0008] Barik, A. , J. H. Thompson , M. Seltzer , N. Ghitani , and A. T. Chesler . 2018. “A Brainstem‐Spinal Circuit Controlling Nocifensive Behavior.” Neuron 100: 1491–1503.e3. 30449655 10.1016/j.neuron.2018.10.037

[ejn70275-bib-0009] Baron, R. , A. H. Dickenson , M. Calvo , S. D. Dib‐Hajj , and D. L. Bennett . 2023. “Maximizing Treatment Efficacy Through Patient Stratification in Neuropathic Pain Trials.” Nature Reviews. Neurology 19: 53–64. 36400867 10.1038/s41582-022-00741-7

[ejn70275-bib-0010] Barthas, F. , J. Sellmeijer , S. Hugel , E. Waltisperger , M. Barrot , and I. Yalcin . 2015. “The Anterior Cingulate Cortex Is a Critical Hub for Pain‐Induced Depression.” Biological Psychiatry 77: 236–245. 25433903 10.1016/j.biopsych.2014.08.004

[ejn70275-bib-0011] Basbaum, A. I. , D. M. Bautista , G. Scherrer , and D. Julius . 2009. “Cellular and Molecular Mechanisms of Pain.” Cell 139: 267–284. 19837031 10.1016/j.cell.2009.09.028 PMC2852643

[ejn70275-bib-0012] Benarroch, E. 2022. “What Are the Interactions Between the Midbrain Dopamine System in Pain?” Neurology 98: 274–278. 35165154 10.1212/WNL.0000000000013253

[ejn70275-bib-0013] Benarroch, E. E. 2019. “Insular Cortex.” Neurology 93: 932–938. 31645470 10.1212/WNL.0000000000008525

[ejn70275-bib-0014] Bhattacherjee, A. , C. Zhang , B. R. Watson , M. N. Djekidel , J. R. Moffitt , and Y. Zhang . 2023. “Spatial Transcriptomics Reveals the Distinct Organization of Mouse Prefrontal Cortex and Neuronal Subtypes Regulating Chronic Pain.” Nature Neuroscience 26: 1880–1893. 37845544 10.1038/s41593-023-01455-9 PMC10620082

[ejn70275-bib-0015] Bliss, T. V. P. , G. L. Collingridge , B.‐K. Kaang , and M. Zhuo . 2016. “Synaptic Plasticity in the Anterior Cingulate Cortex in Acute and Chronic Pain.” Nature Reviews Neuroscience 17: 485–496. 27307118 10.1038/nrn.2016.68

[ejn70275-bib-0016] Borsook, D. , C. Linnman , V. Faria , A. M. Strassman , L. Becerra , and I. Elman . 2016. “Reward Deficiency and Anti‐Reward in Pain Chronification.” Neuroscience and Biobehavioral Reviews 68: 282–297. 27246519 10.1016/j.neubiorev.2016.05.033

[ejn70275-bib-0017] Bravo, L. , M. Llorca‐Torralba , I. Suárez‐Pereira , and E. Berrocoso . 2020. “Pain in Neuropsychiatry: Insights From Animal Models.” Neuroscience and Biobehavioral Reviews 115: 96–115. 32437745 10.1016/j.neubiorev.2020.04.029

[ejn70275-bib-0018] Bushnell, M. C. , G. H. Duncan , R. K. Hofbauer , B. Ha , J.‐I. Chen , and B. Carrier . 1999. “Pain Perception: Is There a Role for Primary Somatosensory Cortex?” Proceedings of the National Academy of Sciences 96: 7705–7709. 10.1073/pnas.96.14.7705 PMC33605 10393884

[ejn70275-bib-0019] Campos, C. A. , A. J. Bowen , C. W. Roman , and R. D. Palmiter . 2018. “Encoding of Danger by Parabrachial CGRP Neurons.” Nature 555: 617–622. 29562230 10.1038/nature25511 PMC6129987

[ejn70275-bib-0020] Caputi, A. , S. Melzer , M. Michael , and H. Monyer . 2013. “The Long and Short of GABAergic Neurons.” Current Opinion in Neurobiology 23: 179–186. 23394773 10.1016/j.conb.2013.01.021

[ejn70275-bib-0021] Castellanos, J. P. , C. Woolley , K. A. Bruno , F. Zeidan , A. Halberstadt , and T. Furnish . 2020. “Chronic Pain and Psychedelics: A Review and Proposed Mechanism of Action.” Regional Anesthesia and Pain Medicine 45: 486–494. 32371500 10.1136/rapm-2020-101273

[ejn70275-bib-0022] Che, T. , and B. L. Roth . 2023. “Molecular Basis of Opioid Receptor Signaling.” Cell 186: 5203–5219. 37995655 10.1016/j.cell.2023.10.029 PMC10710086

[ejn70275-bib-0023] Chen, T. , W. Taniguchi , Q. Chen , et al. 2018. “Top‐down Descending Facilitation of Spinal Sensory Excitatory Transmission from the Anterior Cingulate Cortex.” Nature Communications 9: 1886. 10.1038/s41467-018-04309-2 PMC5951839 29760484

[ejn70275-bib-0024] Chiang, M. C. , A. Bowen , L. A. Schier , D. Tupone , O. Uddin , and M. M. Heinricher . 2019. “Parabrachial Complex: A Hub for Pain and Aversion.” Journal of Neuroscience 39: 8225–8230. 31619491 10.1523/JNEUROSCI.1162-19.2019 PMC6794922

[ejn70275-bib-0025] Coghill, R. C. 2020. “The Distributed Nociceptive System: A Framework for Understanding Pain.” Trends in Neurosciences 43: 780–794. 32800534 10.1016/j.tins.2020.07.004 PMC7530033

[ejn70275-bib-0026] Cohen, S. P. , L. Vase , and W. M. Hooten . 2021. “Chronic Pain: An Update on Burden, Best Practices, and New Advances.” Lancet 397: 2082–2097. 34062143 10.1016/S0140-6736(21)00393-7

[ejn70275-bib-0027] Corder, G. , B. Ahanonu , B. F. Grewe , D. Wang , M. J. Schnitzer , and G. Scherrer . 2019. “An Amygdalar Neural Ensemble That Encodes the Unpleasantness of Pain.” Science 363: 276–281. 30655440 10.1126/science.aap8586 PMC6450685

[ejn70275-bib-0028] Corder, G. , D. C. Castro , M. R. Bruchas , and G. Scherrer . 2018. “Endogenous and Exogenous Opioids in Pain.” Annual Review of Neuroscience 41: 453–473. 10.1146/annurev-neuro-080317-061522 PMC6428583 29852083

[ejn70275-bib-0029] Costa, A. R. , I. Tavares , and I. Martins . 2023. “How Do Opioids Control Pain Circuits in the Brainstem During Opioid‐Induced Disorders and in Chronic Pain? Implications for the Treatment of Chronic Pain.” Pain 165: 324–336. 37578500 10.1097/j.pain.0000000000003026

[ejn70275-bib-0030] Crawford, L. , E. Mills , N. Meylakh , P. M. Macey , V. G. Macefield , and L. A. Henderson . 2023. “Brain Activity Changes Associated With Pain Perception Variability.” Cerebral Cortex 33: 4145–4155. 36069972 10.1093/cercor/bhac332

[ejn70275-bib-0031] Cristino, L. , T. Bisogno , and V. Di Marzo . 2020. “Cannabinoids and the Expanded Endocannabinoid System in Neurological Disorders.” Nature Reviews. Neurology 16: 9–29. 31831863 10.1038/s41582-019-0284-z

[ejn70275-bib-0032] De Preter, C. C. , and M. M. Heinricher . 2024. “The ‘In's and Out's’ of Descending Pain Modulation From the Rostral Ventromedial Medulla.” Trends in Neurosciences 47: 447–460. 38749825 10.1016/j.tins.2024.04.006 PMC11168876

[ejn70275-bib-0033] Dowell, D. , K. R. Ragan , C. M. Jones , G. T. Baldwin , and R. Chou . 2022. “CDC Clinical Practice Guideline for Prescribing Opioids for Pain — United States, 2022.” MMWR ‐ Recommendations and Reports 71: 1–95. 10.15585/mmwr.rr7103a1 PMC9639433 36327391

[ejn70275-bib-0034] Elman, I. , and D. Borsook . 2016. “Common Brain Mechanisms of Chronic Pain and Addiction.” Neuron 89: 11–36. 26748087 10.1016/j.neuron.2015.11.027

[ejn70275-bib-0035] Erritzoe, D. , C. Timmermann , K. Godfrey , et al. 2024. “Exploring Mechanisms of Psychedelic Action Using Neuroimaging.” Nature Mental Health 2: 141–153.

[ejn70275-bib-0036] Finnerup, N. B. , R. Kuner , and T. S. Jensen . 2021. “Neuropathic Pain: From Mechanisms to Treatment.” Physiological Reviews 101: 259–301. 32584191 10.1152/physrev.00045.2019

[ejn70275-bib-0037] Gaborit, M. , and D. Massotte . 2023. “Therapeutic Potential of Opioid Receptor Heteromers in Chronic Pain and Associated Comorbidities.” British Journal of Pharmacology 180: 994–1013. 34883528 10.1111/bph.15772

[ejn70275-bib-0038] Gélébart, J. , L. Garcia‐Larrea , and M. Frot . 2023. “Amygdala and Anterior Insula Control the Passage From Nociception to Pain.” Cerebral Cortex 33: 3538–3547. 35965070 10.1093/cercor/bhac290

[ejn70275-bib-0039] Gilam, G. , J. J. Gross , T. D. Wager , F. J. Keefe , and S. C. Mackey . 2020. “What Is the Relationship between Pain and Emotion? Bridging Constructs and Communities.” Neuron 107: 17–21. 32562660 10.1016/j.neuron.2020.05.024 PMC7578761

[ejn70275-bib-0040] Goel, A. , Y. Rai , S. Sivadas , et al. 2023. “Use of Psychedelics for Pain: A Scoping Review.” Anesthesiology 139: 523–536. 37698433 10.1097/ALN.0000000000004673

[ejn70275-bib-0041] Gogolla, N. 2017. “The insular cortex.” Current Biology 27: R580–R586. 28633023 10.1016/j.cub.2017.05.010

[ejn70275-bib-0042] Gu, X. , Y. Z. Zhang , J. J. O'Malley , C. C. De Preter , M. Penzo , and M. A. Hoon . 2023. “Neurons in the Caudal Ventrolateral Medulla Mediate Descending Pain Control.” Nature Neuroscience 26: 594–605. 36894654 10.1038/s41593-023-01268-w PMC11114367

[ejn70275-bib-0043] Han, S. , M. T. Soleiman , M. E. Soden , L. S. Zweifel , and R. D. Palmiter . 2015. “Elucidating an Affective Pain Circuit that Creates a Threat Memory.” Cell 162: 363–374. 26186190 10.1016/j.cell.2015.05.057 PMC4512641

[ejn70275-bib-0044] Heinricher, M. M. , I. Tavares , J. L. Leith , and B. M. Lumb . 2009. “Descending Control of Nociception: Specificity, Recruitment and Plasticity.” Brain Research Reviews 60: 214–225. 19146877 10.1016/j.brainresrev.2008.12.009 PMC2894733

[ejn70275-bib-0045] Hirschberg, S. , Y. Li , A. Randall , E. J. Kremer , and A. E. Pickering . 2017. “Functional Dichotomy in Spinal‐vs Prefrontal‐Projecting Locus Coeruleus Modules Splits Descending Noradrenergic Analgesia From Ascending Aversion and Anxiety in Rats.” eLife 6: 1–26. 10.7554/eLife.29808 PMC5653237 29027903

[ejn70275-bib-0046] Hu, H. , Y. Cui , and Y. Yang . 2020. “Circuits and Functions of the Lateral Habenula in Health and in Disease.” Nature Reviews Neuroscience 21: 277–295. 32269316 10.1038/s41583-020-0292-4

[ejn70275-bib-0047] Johnson, M. I. , C. A. Paley , T. E. Howe , and K. A. Sluka . 2015. “Transcutaneous Electrical Nerve Stimulation for Acute Pain.” Cochrane Database of Systematic Reviews 2021. 10.1002/14651858.CD006142.pub3 PMC8094447 26075732

[ejn70275-bib-0048] Journée, S. H. , V. P. Mathis , C. Fillinger , P. Veinante , and I. Yalcin . 2023. “Janus Effect of the Anterior Cingulate Cortex: Pain and Emotion.” Neuroscience and Biobehavioral Reviews 153: 105362. 37595650 10.1016/j.neubiorev.2023.105362

[ejn70275-bib-0049] Kaplan, C. M. , E. Kelleher , A. Irani , A. Schrepf , D. J. Clauw , and S. E. Harte . 2024. “Deciphering Nociplastic Pain: Clinical Features, Risk Factors and Potential Mechanisms.” Nature Reviews Neurology 20: 347–363. 38755449 10.1038/s41582-024-00966-8

[ejn70275-bib-0050] Kim, J.‐H. , G. Gangadharan , J. Byun , E.‐J. Choi , C. J. Lee , and H.‐S. Shin . 2018. “Yin‐and‐Yang Bifurcation of Opioidergic Circuits for Descending Analgesia at the Midbrain of the Mouse.” Proceedings of the National Academy of Sciences 115: 11078–11083. 10.1073/pnas.1806082115 PMC6205495 30297409

[ejn70275-bib-0051] Klit, H. , N. B. Finnerup , and T. S. Jensen . 2009. “Central Post‐Stroke Pain: Clinical Characteristics, Pathophysiology, and Management.” Lancet Neurology 8: 857–868. 19679277 10.1016/S1474-4422(09)70176-0

[ejn70275-bib-0052] Knotkova, H. , C. Hamani , E. Sivanesan , et al. 2021. “Neuromodulation for Chronic Pain.” Lancet 397: 2111–2124. 34062145 10.1016/S0140-6736(21)00794-7

[ejn70275-bib-0053] Kohoutová, L. , L. Y. Atlas , C. Büchel , et al. 2022. “Individual Variability in Brain Representations of Pain.” Nature Neuroscience 25: 749–759. 35637368 10.1038/s41593-022-01081-x PMC9435464

[ejn70275-bib-0054] Kohro, Y. , T. Matsuda , K. Yoshihara , et al. 2020. “Spinal Astrocytes in Superficial Laminae Gate Brainstem Descending Control of Mechanosensory Hypersensitivity.” Nature Neuroscience 23: 1376–1387. 33020652 10.1038/s41593-020-00713-4

[ejn70275-bib-0055] Krebs, E. E. , A. Gravely , S. Nugent , et al. 2018. “Effect of Opioid vs Nonopioid Medications on Pain‐Related Function in Patients With Chronic Back Pain or Hip or Knee Osteoarthritis Pain.” JAMA 319: 872–882. 29509867 10.1001/jama.2018.0899 PMC5885909

[ejn70275-bib-0056] Kremer, M. , I. Yalcin , Y. Goumon , et al. 2018. “A Dual Noradrenergic Mechanism for the Relief of Neuropathic Allodynia by the Antidepressant Drugs Duloxetine and Amitriptyline.” Journal of Neuroscience 38: 9934–9954. 30249798 10.1523/JNEUROSCI.1004-18.2018 PMC6596240

[ejn70275-bib-0057] Kuner, R. 2010. “Central Mechanisms of Pathological Pain.” Nature Medicine 16: 1258–1266. 10.1038/nm.2231 20948531

[ejn70275-bib-0058] Kuner, R. , and H. Flor . 2017. “Structural Plasticity and Reorganisation in Chronic Pain.” Nature Reviews Neuroscience 18: 20–30. 10.1038/nrn.2016.162 27974843

[ejn70275-bib-0059] Kuner, R. , and T. Kuner . 2021. “Cellular Circuits in the Brain and Their Modulation in Acute and Chronic Pain.” Physiological Reviews 101: 213–258. 32525759 10.1152/physrev.00040.2019

[ejn70275-bib-0060] Lau, B. K. , B. L. Winters , and C. W. Vaughan . 2020. “Opioid Presynaptic Disinhibition of the Midbrain Periaqueductal Grey Descending Analgesic Pathway.” British Journal of Pharmacology 177: 2320–2332. 31971607 10.1111/bph.14982 PMC7174888

[ejn70275-bib-0061] Lei, J. , L.‐L. Tang , and H.‐J. You . 2024. “Pathological Pain: Non‐Motor Manifestations in Parkinson Disease and Its Treatment.” Neuroscience and Biobehavioral Reviews 161: 105646. 38569983 10.1016/j.neubiorev.2024.105646

[ejn70275-bib-0062] Leknes, S. , and I. Tracey . 2008. “A Common Neurobiology for Pain and Pleasure.” Nature Reviews Neuroscience 9: 314–320. 18354400 10.1038/nrn2333

[ejn70275-bib-0063] Liang, S.‐H. , W.‐J. Zhao , J.‐B. Yin , et al. 2020. “A Neural Circuit From Thalamic Paraventricular Nucleus to Central Amygdala for the Facilitation of Neuropathic Pain.” Journal of Neuroscience 40: 7837–7854. 32958568 10.1523/JNEUROSCI.2487-19.2020 PMC7548696

[ejn70275-bib-0064] Lim, S. Y. , and P. Cengiz . 2022. “Opioid Tolerance and Opioid‐Induced Hyperalgesia: Is TrkB Modulation a Potential Pharmacological Solution?” Neuropharmacology 220: 109260. 36165856 10.1016/j.neuropharm.2022.109260

[ejn70275-bib-0065] Malcangio, M. , and G. Sideris‐Lampretsas . 2025. “How Microglia Contribute to the Induction and Maintenance of Neuropathic Pain.” Nature Reviews. Neuroscience 26: 263–275. 40128335 10.1038/s41583-025-00914-5

[ejn70275-bib-0066] Martins, I. , P. Carvalho , M. G. de Vries , et al. 2015. “Increased Noradrenergic Neurotransmission to a Pain Facilitatory Area of the Brain Is Implicated in Facilitation of Chronic Pain.” Anesthesiology 123: 642–653. 26146901 10.1097/ALN.0000000000000749

[ejn70275-bib-0067] Martins, I. , S. Costa‐Araújo , J. Fadel , S. P. Wilson , D. Lima , and I. Tavares . 2010. “Reversal of Neuropathic Pain by HSV‐1‐Mediated Decrease of Noradrenaline in a Pain Facilitatory Area of the Brain.” Pain 151: 137–145. 20637543 10.1016/j.pain.2010.06.027

[ejn70275-bib-0068] Martins, I. , and I. Tavares . 2017. “Reticular Formation and Pain: The Past and the Future.” Frontiers in Neuroanatomy 11: 1–14. 28725185 10.3389/fnana.2017.00051 PMC5497058

[ejn70275-bib-0069] Millan, M. J. 2002. “Descending Control of Pain.” Progress in Neurobiology 66: 355–474. 12034378 10.1016/s0301-0082(02)00009-6

[ejn70275-bib-0070] Mills, E. P. , F. Di Pietro , Z. Alshelh , et al. 2018. “Brainstem Pain‐Control Circuitry Connectivity in Chronic Neuropathic Pain.” Journal of Neuroscience 38: 465–473. 29175957 10.1523/JNEUROSCI.1647-17.2017 PMC6596109

[ejn70275-bib-0071] Mogil, J. S. 2020. “Qualitative Sex Differences in Pain Processing: Emerging Evidence of a Biased Literature.” Nature Reviews Neuroscience 21: 353–365. 32440016 10.1038/s41583-020-0310-6

[ejn70275-bib-0072] Morales, P. , and N. Jagerovic . 2020. “Novel Approaches and Current Challenges With Targeting the Endocannabinoid System.” Expert Opinion on Drug Discovery 15: 917–930. 32336154 10.1080/17460441.2020.1752178 PMC7502221

[ejn70275-bib-0073] Navratilova, E. , C. W. Atcherley , and F. Porreca . 2015. “Brain Circuits Encoding Reward From Pain Relief.” Trends in Neurosciences 38: 741–750. 26603560 10.1016/j.tins.2015.09.003 PMC4752429

[ejn70275-bib-0074] Navratilova, E. , and F. Porreca . 2014. “Reward and Motivation in Pain and Pain Relief.” Nature Neuroscience 17: 1304–1312. 25254980 10.1038/nn.3811 PMC4301417

[ejn70275-bib-0075] Navratilova, E. , C. Qu , G. Ji , et al. 2023. “Opposing Effects on Descending Control of Nociception by Mu and Kappa Opioid Receptors in the Anterior Cingulate Cortex.” Anesthesiology 140: 272–283. 10.1097/ALN.0000000000004773 PMC11466009 37725756

[ejn70275-bib-0076] Neugebauer, V. , M. Mazzitelli , B. Cragg , G. Ji , E. Navratilova , and F. Porreca . 2020. “Amygdala, Neuropeptides, and Chronic Pain‐Related Affective Behaviors.” Neuropharmacology 170: 108052. 32188569 10.1016/j.neuropharm.2020.108052 PMC7214122

[ejn70275-bib-0077] Neugebauer, V. , P. Presto , V. Yakhnitsa , N. Antenucci , B. Mendoza , and G. Ji . 2023. “Pain‐Related Cortico‐Limbic Plasticity and Opioid Signaling.” Neuropharmacology 231: 109510. 36944393 10.1016/j.neuropharm.2023.109510 PMC10585936

[ejn70275-bib-0078] Nguyen, E. , J. G. Grajales‐Reyes , R. W. Gereau , and S. E. Ross . 2023. “Cell Type‐Specific Dissection of Sensory Pathways Involved in Descending Modulation.” Trends in Neurosciences 46: 539–550. 37164868 10.1016/j.tins.2023.04.002 PMC10836406

[ejn70275-bib-0079] O'Connell, N. E. , L. Marston , S. Spencer , L. H. DeSouza , and B. M. Wand . 2018. “Non‐Invasive Brain Stimulation Techniques for Chronic Pain.” Cochrane Database of Systematic Reviews 2018: 1–284. 10.1002/14651858.CD008208.pub4 PMC7039253 29547226

[ejn70275-bib-0080] Ossipov, M. H. , G. O. Dussor , and F. Porreca . 2010. “Central Modulation of Pain.” Journal of Clinical Investigation 120: 3779–3787. 21041960 10.1172/JCI43766 PMC2964993

[ejn70275-bib-0081] Palmiter, R. D. 2018. “The Parabrachial Nucleus: CGRP Neurons Function as a General Alarm.” Trends in Neurosciences 41: 280–293. 29703377 10.1016/j.tins.2018.03.007 PMC5929477

[ejn70275-bib-0082] Palmiter, R. D. 2024. “Parabrachial Neurons Promote Nociplastic Pain.” Trends in Neurosciences 47: 722–735. 39147688 10.1016/j.tins.2024.07.002

[ejn70275-bib-0083] Pertovaara, A. 2013. “The Noradrenergic Pain Regulation System: A Potential Target for Pain Therapy.” European Journal of Pharmacology 716: 2–7. 23500194 10.1016/j.ejphar.2013.01.067

[ejn70275-bib-0084] Poe, G. R. , S. Foote , O. Eschenko , et al. 2020. “Locus Coeruleus: A New Look at the Blue Spot.” Nature Reviews Neuroscience 21: 644–659. 32943779 10.1038/s41583-020-0360-9 PMC8991985

[ejn70275-bib-0085] Raja, S. N. , D. B. Carr , M. Cohen , et al. 2020. “The Revised International Association for the Study of Pain Definition of Pain: Concepts, Challenges, and Compromises.” Pain 161: 1976–1982. 32694387 10.1097/j.pain.0000000000001939 PMC7680716

[ejn70275-bib-0086] Raver, C. , O. Uddin , Y. Ji , et al. 2020. “An Amygdalo‐Parabrachial Pathway Regulates Pain Perception and Chronic Pain.” Journal of Neuroscience 40: 3424–3442. 32217613 10.1523/JNEUROSCI.0075-20.2020 PMC7178908

[ejn70275-bib-0087] Rosner, J. , D. C. de Andrade , K. D. Davis , et al. 2023. “Central Neuropathic Pain.” Nature Reviews Disease Primers 9: 73. 10.1038/s41572-023-00484-9 PMC11329872 38129427

[ejn70275-bib-0088] Russo, S. J. , and E. J. Nestler . 2013. “The Brain Reward Circuitry in Mood Disorders.” Nature Reviews Neuroscience 14: 609–625. 23942470 10.1038/nrn3381 PMC3867253

[ejn70275-bib-0089] Sadler, K. E. , J. S. Mogil , and C. L. Stucky . 2022. “Innovations and Advances in Modelling and Measuring Pain in Animals.” Nature Reviews Neuroscience 23: 70–85. 34837072 10.1038/s41583-021-00536-7 PMC9098196

[ejn70275-bib-0090] Sandkühler, J. 2009. “Models and Mechanisms of Hyperalgesia and Allodynia.” Physiological Reviews 89: 707–758. 19342617 10.1152/physrev.00025.2008

[ejn70275-bib-0091] Serafini, R. A. , K. D. Pryce , and V. Zachariou . 2020. “The Mesolimbic Dopamine System in Chronic Pain and Associated Affective Comorbidities.” Biological Psychiatry 87: 64–73. 31806085 10.1016/j.biopsych.2019.10.018 PMC6954000

[ejn70275-bib-0092] Shackman, A. J. , T. V. Salomons , H. A. Slagter , A. S. Fox , J. J. Winter , and R. J. Davidson . 2011. “The Integration of Negative Affect, Pain and Cognitive Control in the Cingulate Cortex.” Nature Reviews Neuroscience 12: 154–167. 21331082 10.1038/nrn2994 PMC3044650

[ejn70275-bib-0093] Shiers, S. , and T. J. Price . 2020. “Molecular, Circuit, and Anatomical Changes in the Prefrontal Cortex in Chronic Pain.” Pain 161: 1726–1729. 32701833 10.1097/j.pain.0000000000001897 PMC7575617

[ejn70275-bib-0094] Shine, J. M. , L. D. Lewis , D. D. Garrett , and K. Hwang . 2023. “The Impact of the Human Thalamus on Brain‐Wide Information Processing.” Nature Reviews Neuroscience 24: 416–430. 37237103 10.1038/s41583-023-00701-0 PMC10970713

[ejn70275-bib-0095] Siegel, J. S. , S. Subramanian , D. Perry , et al. 2024. “Psilocybin Desynchronizes the Human Brain.” Nature 632: 131–138. 39020167 10.1038/s41586-024-07624-5 PMC11291293

[ejn70275-bib-0096] Sirucek, L. , R. P. Ganley , H. U. Zeilhofer , and P. Schweinhardt . 2023. “Diffuse Noxious Inhibitory Controls and Conditioned Pain Modulation: A Shared Neurobiology Within the Descending Pain Inhibitory System?” Pain 164: 463–468. 36017879 10.1097/j.pain.0000000000002719 PMC9916052

[ejn70275-bib-0097] Soliman, N. , X. Moisset , M. C. Ferraro , et al. 2025. “Pharmacotherapy and Non‐Invasive Neuromodulation for Neuropathic Pain: A Systematic Review and Meta‐Analysis.” Lancet Neurology 24: 413–428. 40252663 10.1016/S1474-4422(25)00068-7

[ejn70275-bib-0098] Stegemann, A. , S. Liu , O. A. Retana Romero , et al. 2023. “Prefrontal Engrams of Long‐Term Fear Memory Perpetuate Pain Perception.” Nature Neuroscience 26: 820–829. 37024573 10.1038/s41593-023-01291-x PMC10166861

[ejn70275-bib-0099] Suárez‐Pereira, I. , M. Llorca‐Torralba , L. Bravo , C. Camarena‐Delgado , C. Soriano‐Mas , and E. Berrocoso . 2022. “The Role of the Locus Coeruleus in Pain and Associated Stress‐Related Disorders.” Biological Psychiatry 91: 786–797. 35164940 10.1016/j.biopsych.2021.11.023

[ejn70275-bib-0100] Sullere, S. , A. Kunczt , and D. S. McGehee . 2023. “A Cholinergic Circuit That Relieves Pain Despite Opioid Tolerance.” Neuron 111: 3414–3434.e15. 37734381 10.1016/j.neuron.2023.08.017 PMC10843525

[ejn70275-bib-0101] Tan, L. L. , and R. Kuner . 2021. “Neocortical Circuits in Pain and Pain Relief.” Nature Reviews Neuroscience 22: 458–471. 34127843 10.1038/s41583-021-00468-2

[ejn70275-bib-0102] Tan, L. L. , M. J. Oswald , C. Heinl , et al. 2019. “Gamma Oscillations in Somatosensory Cortex Recruit Prefrontal and Descending Serotonergic Pathways in Aversion and Nociception.” Nature Communications 10: 983. 10.1038/s41467-019-08873-z PMC6395755 30816113

[ejn70275-bib-0103] Tan, L. L. , P. Pelzer , C. Heinl , et al. 2017. “A Pathway From Midcingulate Cortex to Posterior Insula Gates Nociceptive Hypersensitivity.” Nature Neuroscience 20: 1591–1601. 28920932 10.1038/nn.4645

[ejn70275-bib-0104] Tang, Q.‐Q. , Y. Wu , Q. Tao , et al. 2023. “Direct Paraventricular Thalamus‐Basolateral Amygdala Circuit Modulates Neuropathic Pain and Emotional Anxiety.” Neuropsychopharmacology 49: 455–466. 37848732 10.1038/s41386-023-01748-4 PMC10724280

[ejn70275-bib-0105] Tappe‐Theodor, A. , and R. Kuner . 2019. “A Common Ground for Pain and Depression.” Nature Neuroscience 22: 1612–1614. 31455879 10.1038/s41593-019-0499-8

[ejn70275-bib-0106] Torres‐Rodriguez, J. M. , T. D. Wilson , S. Singh , et al. 2023. “The Parabrachial to Central Amygdala Pathway Is Critical to Injury‐Induced Pain Sensitization in Mice.” Neuropsychopharmacology 49: 508–520. 37542159 10.1038/s41386-023-01673-6 PMC10789863

[ejn70275-bib-0107] Traynor, J. R. , and J. A. Moron . 2023. “Opioid Research in the Time of the Opioid Crisis.” British Journal of Pharmacology 180: 793–796. 36813266 10.1111/bph.16043 PMC11071141

[ejn70275-bib-0108] Treede, R.‐D. , W. Rief , A. Barke , et al. 2019. “Chronic Pain as a Symptom or a Disease: The IASP Classification of Chronic Pain for the International Classification of Diseases (ICD‐11).” Pain 160: 19–27. 30586067 10.1097/j.pain.0000000000001384

[ejn70275-bib-0109] van Elk, M. , and D. B. Yaden . 2022. “Pharmacological, Neural, and Psychological Mechanisms Underlying Psychedelics: A Critical Review.” Neuroscience and Biobehavioral Reviews 140: 104793. 35878791 10.1016/j.neubiorev.2022.104793

[ejn70275-bib-0110] Varga, B. R. , J. M. Streicher , and S. Majumdar . 2023. “Strategies Towards Safer Opioid Analgesics—A Review of Old and Upcoming Targets.” British Journal of Pharmacology 180: 975–993. 34826881 10.1111/bph.15760 PMC9133275

[ejn70275-bib-0111] Vogt, B. A. 2005. “Pain and Emotion Interactions in Subregions of the Cingulate Gyrus.” Nature Reviews Neuroscience 6: 533–544. 15995724 10.1038/nrn1704 PMC2659949

[ejn70275-bib-0112] Wang, L.‐H. , W.‐Q. Ding , and Y.‐G. Sun . 2022. “Spinal Ascending Pathways for Somatosensory Information Processing.” Trends in Neurosciences 45: 594–607. 35701247 10.1016/j.tins.2022.05.005

[ejn70275-bib-0113] Wiech, K. 2016. “Deconstructing the Sensation of Pain: The Influence of Cognitive Processes on Pain Perception.” Science 354: 584–587. 27811269 10.1126/science.aaf8934

[ejn70275-bib-0114] Wilson, T. D. , S. Valdivia , A. Khan , et al. 2019. “Dual and Opposing Functions of the Central Amygdala in the Modulation of Pain.” Cell Reports 29: 332–346.e5. 31597095 10.1016/j.celrep.2019.09.011 PMC6816228

[ejn70275-bib-0115] Winters, B. L. , B. K. Lau , and C. W. Vaughan . 2022. “Cannabinoids and Opioids Differentially Target Extrinsic and Intrinsic GABAergic Inputs onto the Periaqueductal Grey Descending Pathway.” Journal of Neuroscience 42: JN‐RM‐0997‐22. 10.1523/JNEUROSCI.0997-22.2022 PMC9581564 36414010

[ejn70275-bib-0116] Zhou, W. , Y. Jin , Q. Meng , et al. 2019. “A Neural Circuit for Comorbid Depressive Symptoms in Chronic Pain.” Nature Neuroscience 22: 1649–1658. 31451801 10.1038/s41593-019-0468-2

